# Developing an mHealth Intervention to Reduce COVID-19–Associated Psychological Distress Among Health Care Workers in Nigeria: Protocol for a Design and Feasibility Study

**DOI:** 10.2196/36174

**Published:** 2022-11-16

**Authors:** Adesanmi Akinsulore, Olutayo Aloba, Olakunle Oginni, Ibidunni Oloniniyi, Olanrewaju Ibigbami, Champion Tobi Seun-Fadipe, Tolulope Opakunle, Afolabi Muyiwa Owojuyigbe, Olushola Olibamoyo, Boladale Mapayi, Victor Ogbonnaya Okorie, Abiodun Olugbenga Adewuya

**Affiliations:** 1 Department of Mental Health Faculty of Clinical Sciences Obafemi Awolowo University Ile-Ife Nigeria; 2 Department of Mental Health Obafemi Awolowo University Teaching Hospitals Complex Ile-Ife Nigeria; 3 Nottinghamshire Healthcare NHS Trust Nottinghamshire United Kingdom; 4 Department of Mental Health State Specialist Hospital Osogbo Nigeria; 5 Department of Anaesthesia and Intensive Care Obafemi Awolowo University Ile-Ife Nigeria; 6 Department of Anaesthesia and Intensive Care Obafemi Awolowo University Teaching Hospitals Complex Ile-Ife Nigeria; 7 Department of Behavioural Medicine Lagos State University College of Medicine Lagos Nigeria; 8 Department of Agricultural Extension and Rural Development Obafemi Awolowo University Ile-Ife Nigeria

**Keywords:** COVID-19, psychological distress, Nigeria, health care workers, mental health, well-being, pandemic, mHealth, mobile health, digital health intervention, health intervention, health care, smartphone, mobile phone

## Abstract

**Background:**

Globally, COVID-19–related psychological distress is seriously eroding health care workers’ mental health and well-being, especially in low-income countries like Nigeria. The use of mobile health (mHealth) interventions is now increasingly recognized as an innovative approach that may improve mental health and well-being. This project aims to develop an mHealth psychological intervention (mPsyI) to reduce COVID-19–related psychological distress among health care workers in Nigeria.

**Objective:**

Our objective is to present a study protocol to determine the level of COVID-19–related psychological distress among health care workers in Nigeria; explore health care workers’ experience of COVID-19–related psychological distress; develop and pilot test mPsyI to reduce this distress; and assess the feasibility of this intervention (such as usability, engagement, and satisfaction).

**Methods:**

A mixed (quantitative and qualitative) methods approach is used in which health care workers will be recruited from 2 tertiary health care facilities in southwest Nigeria. The study is divided into 4 phases based on the study objectives. Phase 1 involves a quantitative survey to assess the type and levels of psychosocial distress. Phase 2 collects qualitative data on psychosocial distress among health care workers. Phase 3 involves development of the mHealth-based psychological intervention, and phase 4 is a mixed methods study to assess the feasibility and acceptability of the intervention.

**Results:**

This study was funded in November 2020 by the Global Effort on COVID-19 Health Research, and collection of preliminary baseline data started in July 2021.

**Conclusions:**

This is the first study to report the development of an mHealth-based intervention to reduce COVID-19–related psychological distress among health care workers in Nigeria. Using a mixed methods design in this study can potentially facilitate the adaptation of an evidence-based treatment method that is culturally sensitive and cost-effective for the management of COVID-19–related psychological distress among health care workers in Nigeria.

**International Registered Report Identifier (IRRID):**

DERR1-10.2196/36174

## Introduction

Globally, medical doctors and nurses have been praised for their dedication in providing care for those affected with SARS-CoV-2, responsible for the ongoing COVID-19 pandemic. The frontline occupied by medical doctors and nurses in the fight against COVID-19 has had a heavy toll on their mental health [1]. A high prevalence of COVID-19–related psychological distress among medical doctors and nurses has been reported in Nigeria [2,3].

Psychological interventions targeting medical doctors and nurses are very important, as they comprise a high-risk group for COVID-19–related psychological distress [4]. Moreover, mobile health (mHealth) interventions are increasingly seen by some experts as a game changer in the context of solutions to mental health and well-being challenges [5,6]. mHealth describes various health care practices and delivery based on apps or software installed on mobile devices, such as smartphones or phablets, client supervising and monitoring devices, and personal digital assistants [7]. The concept of mHealth also pertains to using these devices to synthesize and store data as well as retrieve and exchange information among those connected to the mHealth platform [8]. The provision of medical and public health services through mHealth is dependent primarily on the mobile phone use of SMS text messaging, voice, and multimedia services [9].

In achieving universal health coverage, mHealth can broaden health care services’ quality and reach and enhance human resources’ capacity [10]. Due to the widespread usage of smartphones, mHealth apps are an increasingly acceptable avenue for implementing interventions for psychological or mental health problems [11]. One significant advantage of the mHealth technology is its capability for periodic sampling and recording the prevailing behaviors and experiences of the users in real time and in natural settings; this is described as ambulatory assessment or experience sampling method [12]. mHealth-based ambulatory assessment can also be applied with psychological or behavior change interventions, a concept described as ecological momentary interventions whereby treatment is provided to subjects in real-time contexts and settings [13]. These treatments can be administered independently or as a supplement to other ongoing treatments. The use of mHealth interventions has been described as a “therapist in the pocket” treatment technique and is extensively perceived to have the capability to transform psychological treatment [14].

Nigeria, the seventh most populous country globally, has an estimated population of 203 million [15] and has the most extensive and fastest-growing mobile phone market on the African continent [16]. Currently, the use of smartphones in Nigeria is approximated at 40 million, and it has been projected to increase to 140 million by the year 2025 [17]. The use of smartphones has been described as universal among Nigerian doctors and nurses [18]. Although a study reported that a significant percentage of Nigerian medical doctors and nurses were not familiar with the term “mHealth,” most are aware of the application of mobile phones in health care and delivery [19]. A systematic review that evaluated mHealth interventions targeted at health care workers reported that these interventions focused primarily on patient data collection during hospital visits, facilitation of communication between health care workers and patients, interactions between health care workers, and public health monitoring [20].

A literature search revealed that no study had described an mHealth-based intervention protocol directly targeted at reducing COVID-19 pandemic–related psychological distress among Nigerian health care workers, specifically medical doctors and nurses. Most of the empirical evidence for the use of mHealth interventions to improve the mental health of health care workers are from the high-income countries such as Spain [21,22]. The authors of an mHealth-based intervention to reduce mental health problems among Spanish health care workers during the COVID-19 pandemic indicated that additional mHealth treatments specifically tailored to improving the mental well-being of health care workers are needed [21]. However, this intervention was mostly beneficial to participants who were also receiving medication and psychotherapy and may reflect the short duration of the intervention—2 weeks. A meta-analysis of randomized clinical trials of psychological interventions delivered through mHealth for anxiety [23] and depressive [24] symptoms in a general population sample reported statistically significant reductions in symptom severity among those exposed to the interventions as compared to the control group.

Due to the impact of the COVID-19 pandemic on the mental health and well-being of health care workers, we acquired funding to develop or adapt and evaluate the feasibility (ie, usability, engagement, satisfaction, acceptability, benefits, and challenges) of an mHealth-based psychological intervention (mPsyI) specifically for medical doctors and nurses in Nigeria. This intervention is a subjectively managed and subjectively guided psychoeducation mobile-based treatment app that does not require the support of a therapist to ease the symptoms of psychological distress (ie, anxiety and depressive symptoms). This paper presents the description of the protocol for a study on developing the mPsyI app, in conformity with the Standard Protocol Item: Recommendations for International Trials (SPIRIT) guidelines [25]. The overall aim of the study is to investigate COVID-19–associated psychosocial distress and evaluate the feasibility of using the mHealth-based intervention in managing this distress among health care workers in Nigeria. The specific objectives are to assess the type and level of psychosocial distress associated with COVID-19 among health workers in Nigeria; explore health care worker’s experience of psychosocial distress associated with COVID-19; develop an mHealth-based guided psychological intervention; and assess the feasibility of the intervention.

## Methods

### Study Setting

The target population comprised doctors and nurses working in the following 2 tertiary hospitals in southwest Nigeria: the Obafemi Awolowo University Teaching Hospital Complex (OAUTHC) in Ile-Ife and the Lagos State University Teaching Hospital (LASUTH) in Lagos. The 2 hospitals were chosen because of ease of access, similar health care delivery structures, and minimal cultural differences. OAUTHC is a Federal Government–owned tertiary hospital with 465 doctors and 887 nurses, and LASUTH is a State Government–owned tertiary hospital with 536 doctors and 987 nurses.

### Design

The study will employ a mixed methods (quantitative and qualitative) approach.

### Recruitment

At the beginning of study phases 1 and 2 (explained below), electronic advertisements including links to the survey were broadcast on professional social media platforms used by health workers in OAUTHC and LASUTH, Nigeria, such as WhatsApp groups, Facebook, email lists, and departmental noticeboards. The advertisements were rebroadcast 3 times a week for the duration of each study period or until the optimal sample size was attained. These were supplemented by physical advertisements placed on departmental noticeboards in the hospital. Participants could follow links in these advertisements to the participant information sheets for both study phases. The sheets contained the aims and scope of the study, the contact details of the principal investigator for further enquiries, and a web-based form to provide contact details for prospective participants in the qualitative studies (to help research assistants contact participants); consent for the web-based survey was obtained by asking participants to select a button indicating this on the survey.

### Sample Size

Phase 1 (quantitative study) comprised 440 nurses and doctors (ie, health care workers), derived using the sample size formula for estimating population proportions using 38.5% as the proportion of health care workers with psychiatric morbidity, based on previous work among Nigerian health care workers [26]. We estimated a minimum sample size of 364 to give a power of 80%, and this was increased by 20% to 440 to allow for incomplete data. Phase 2 (qualitative study) comprised 60 in-depth interviews and 20 key informant interviews and 4 focus group discussions (24 participants). Analyses were carried out alongside data collection to enable us to detect saturation and discontinue subsequent interviews. In phase 4 (mixed methods study), 40 participants will be purposely selected for an in-depth interview.

### Procedure

The study was divided into 4 phases as follows: in the first phase, a quantitative survey will be conducted among health care workers to assess the type and levels of psychosocial distress. The second phase involves qualitative data collection (ie, in-depth interviews, key informant interview, and focus group discussions) among health care workers. In the third phase, a modified Delphi panel comprising a group of experts will be conducted to develop the mHealth-based psychological intervention according to an available evidence-based intervention tool kit [6,27], and the fourth phase is a mixed methods study to assess the feasibility and acceptability of the mHealth-based guided psychological intervention.

#### Phase 1 (Quantitative Survey)

In this phase, doctors and nurses from the 2 hospitals who consented to participate in the study were requested to complete the following measures: The Kessler Psychological Distress Scale [28], the 9-item Patient Health Questionnaire [29], the 7-item Generalized Anxiety Disorder scale [30], the Short Adapted Social Capital Assessment Tool [31], and the Social Connectedness Scale-Revised [32].

These questionnaires were administered physically, and the responses built into electronic forms using REDCap (Version 11.1.2). Only one entry was allowed per participant for the web-based survey. After completing the survey, we asked participants interested in follow-up studies (phase 2) to provide their contact details, which were stored separately from their provided data. To ensure sociocultural relevance, the variables were selected based on previous research among Nigerians, which showed that pandemic-related stress was associated with higher anxiety and depressive symptoms and perceived social support was protective against these adverse mental health outcomes among Nigerians [33].

#### Phase 2 (Qualitative Study)

The aim was to explore health care workers’ experience of COVID-19–associated psychosocial distress and available psychosocial support in their workplace. It comprised 60 in-depth interviews, 20 key informant interviews, and 4 focus group discussions. Previous qualitative studies have recommended a minimum sample size of at least twelve to reach data saturation [34,35]. Through the interviews, we contextualized and understood the experience of psychological distress associated with the COVID-19 pandemic among health care workers; identified protective factors and available psychosocial support at their workplace; and explored the desired features and preferences for an mHealth-based psychological intervention as well as potential barriers to intervention delivery. The in-depth interviews and key informant interviews were conducted through telephone calls and physical meetings (the recommended social distancing measures by the World Health Organization and appropriate personal protective equipment were used). Interview guides were developed, and all interviews were conducted in English, audio recorded, and transcribed for analyses.

#### Phase 3 (Intervention Development)

This phase aims to develop gender-related and culturally sensitive aspects of the mHealth guided psychological intervention. Results from phase 1 will provide quantitative data, and phase 2 will provide qualitative data, such as the expression of psychological distress among Nigerian health care workers. Phase 2 will inform the sociocultural adaptation of available evidence-based psychological interventions [6,27]. Specifically, themes that emerge from phase 2 will be used to contextualize the intervention by changing phrases in the intervention in a more culturally appropriate way and using culturally relevant examples to make it more relevant to the lived experiences of Nigerian health care workers. Both male and female Nigerian voices will be used in the oral or spoken draft of the mPsyI intervention.

A modified Delphi approach (carried until saturation is achieved) will be conducted among a panel of experts in psychiatry, clinical psychology, guidance and counselling, nursing, and computer science. An intervention will be identified based on the World Health Organization collection of low-intensity psychological interventions [6,27]. This intervention will be independently scored for feasibility (ie, usability, engagement, satisfaction, acceptability, benefits, and challenges) using a 5-point scale. The experts will later decide on intervention options based on brevity, cost, the best mode of delivery, and the total number of sessions and then produce an initial draft of the mPsyI intervention. The initial oral or spoken draft of the mPsyI will be presented to end users (ie, health care workers with psychological distress) and facilitators (ie, health care workers without significant psychological distress or professional qualifications in mental health) to assess the practical application, usefulness, and feasibility of the proposed intervention and give feedback via questionnaires (ie, the System Usability Scale and the Mobile App Rating Scale, described below) and informal interviews. Both groups will suggest possible improvements and modifications of the intervention, including the language and contents of the intervention package, during discussions. These suggestions will be returned to the Delphi panel, who will then produce an amended draft of the mPsyI intervention tool kits, including manuals, training guidelines, as well as monitoring and evaluation methods.

#### Phase 4 (Feasibility Assessment)

In this phase, feasibility and pilot test of the mPsyI intervention will be assessed. A total of 8 facilitators (2 doctors and 2 nurses per hospital) will be purposively selected, trained, and supervised by 2 research team members. The facilitators’ training will consist of didactic lectures, clinical demonstrations, and role-plays. Training will be standardized across both study centers with the use of video or audiotapes. Before and after the intervention, end users (ie, medical doctors and nurses) will complete measures used in phase 1. Those with a Kessler Psychological Distress Scale score of 5 and above (being the threshold for significant psychological distress) [28] will be recruited. For 6-8 weeks (depending on the total number of sessions), interventions will be delivered to 40 end users (20 from each hospital)—1 session per week. Previous qualitative studies have recommended a minimum sample size of at least twelve to reach data saturation [34,35].

The trained facilitators will provide weekly support to the end users to make sure that the intervention is being used appropriately. At the end of intervention delivery, the end users and facilitators will test the feasibility of the intervention concerning usability, engagement, satisfaction, acceptability, benefits, and challenges. Additionally, the System Usability Scale [36] and the Mobile App Rating Scale [37] will be used quantitatively to assess user’s experience in terms of intervention acceptability, engagement, satisfaction, and complexity.

### Measures

Before and after the intervention, the Kessler Psychological Distress Scale [28], the 9-item Patient Health Questionnaire [29], and the 7-item Generalized Anxiety Disorder Scale [30] will be used. After administering the intervention, the System Usability Scale [36] will be used to assess the user’s experience in terms of engagement, satisfaction, level of motivation, and complexity of the tool; the Mobile App Rating Scale [37] will be used to assess the acceptability of the tool in terms of engagement, functionality, aesthetics, and information quality. A semistructured interview will be conducted on benefits, challenges, and barriers among end users and facilitators within a week of completing the intervention.

### Data Management and Storage

In phase 1, data will be collected using web-based surveys, exported into SPSS (version 27; IBM Corp) for analyses and stored on a secure passworded laptop, which will be stored on the university premises. Contact details provided were stored separately on a different secure laptop accessible only to the principal investigator, and they will be destroyed immediately after the study. In phases 2 and 4, the audio recordings will be destroyed immediately after transcription, and deidentified transcripts will be saved on a password-protected computer that only the principal investigator and statistician will access. An external hard drive will be used as backup with data encrypted (using full-disk encryption) and stored using a password, and it will be kept in a locked office. Transcripts will only be shared with other members of the research team.

### Statistical Analysis

The recorded interviews will be transcribed verbatim and uploaded into the NVivo 12 for analyses using the framework approach [38]. The framework approach involves 3 interconnected steps that include familiarization with the transcript, deciding initial themes or categories, and summarizing or synthesizing the data [39]. For quantitative data, statistical analyses will be performed using the IBM-SPSS software for Windows (version 27; IBM Corp). Descriptive statistics, frequency distributions, and percentages will be used for categorical variables. For continuous variables, mean, median, standard deviation, percentiles, and ranges will be used. Between-group percentages will be compared with chi-square tests for observed differences, and the student *t* test will be used to determine differences in scores in different groups. Bivariate relationship will be investigated using Pearson correlation, and multiple regression analyses will be used to examine the relationship between psychological distress and the independent variables. Statistical significance will be based on 2-sided tests and set at *P*<.05.

### Ethical Consideration

We obtained ethical approval for the research from the ethics and research committees of OAUTHC (number ERC/2020/10/17) and LASUTH (number LREC/06/10/1528), Nigeria; a favorable ethical opinion was further obtained from the Liverpool School of Tropical Medicine research ethics committee. Informed consent will be obtained from the participants at all phases of the study, and confidentiality will be maintained, as anonymized data will be used for storage and analysis. In testing the mHealth app, only the mental health personnel directly involved in testing the app will have access to data to preserve confidentiality. All identifying information will be excluded before transferring the data to the statisticians for analysis. Participants will also be reassessed midway and at the end of the study to identify those with persistent or increasing distress. Participants with persistent distress in Phase 4 who consent will be referred for more specialized care. Those who do not consent will be provided with contacts they can access for support.

### Dissemination of Knowledge

Findings from the quantitative and qualitative studies in the first and second phases of the project will be used to design the intervention in the third phase of the project. The findings from all phases of the project will be summarized and disseminated to the public via television and radio programs; to stakeholders in hospitals, policymakers, as well as the federal and state ministries of health via webinars; and to the scientific community through local and international conferences and publications in open-access journals. All participants will be invited to provide their contact details to receive summaries of the study results at all study phases.

## Results

Recruitment for phase 1 took 2 months (July to August 2021). The manuscript of data collected in phase 1 titled “psychological distress and associated factors among Nigerian health care workers during COVID-19 pandemic: a cross-sectional study” is under review in the *International Journal of Public Health*. Data collection for phase 2 occurred over 2 months from August through September 2021. Phase 3 lasted for 3 months between October and December 2022, and phase 4 was carried out over 2 months between January and February 2022. Data analysis and scientific reporting are expected to be completed before the end of 2022.

## Discussion

To our knowledge, this is the first study to describe the protocol for the development and evaluation of the feasibility of an mHealth-based intervention to reduce COVID-19 pandemic–related psychological distress among Nigerian medical doctors and nurses. This study hypothesizes that there will be high levels of COVID-19–related psychological distress among health care workers, and the mHealth psychological intervention is a feasible solution for this type of distress among doctors and nurses in Nigeria. Drawing from previous disasters, such as the SARS epidemic and the terrorist attack on September 11, 2001, in the United States, up to 20% of health care workers had stress-related disorders immediately after the events [40]. This is probably because health care workers have to provide care for patients affected by these occurrences, and they also have to navigate their own personal stress and uncertainties [41]. The data obtained in the feasibility study (phase 4) will guide further modifications to the intervention and the potential for a randomized controlled trial later (a potential offshoot of this study). The study will yield insight into the feasibility of providing an mHealth web-based intervention for Nigerian medical doctors and nurses currently experiencing psychological distress due to the COVID-19 pandemic. The results of this study can guide future implementation and promulgation of mHealth-based interventions for other occupational groups in Nigeria. The execution and dissemination of an mHealth evidence-based web-based intervention aimed at improving mental well-being can potentially represent one of the strategies to reduce the mental health gap in low- and middle-income countries, where there is an imbalance between the availability and geographical spread of mental health care specialists and the proportion of those who are experiencing mental health difficulties [5,42].

The exponential increase in the infiltration of internet services and smartphones in low- and middle-income countries can spur the implementation of mHealth-based interventions [43,44]. A plausible advantage of our mHealth intervention is that it might assist in overcoming mental disorder–related stigma, since the end users of this intervention can connect to mental health care services regardless of their location in Nigeria [45]. The longer duration of our intervention may also allow for more time for the manifestation of its therapeutic effects. This mHealth intervention may enhance the quality of life of Nigerian medical doctors and nurses during the ongoing COVID-19 pandemic. Another plausible benefit of this mHealth intervention is the prospect for scalability that will enable it to be available to a greater proportion of Nigerian medical doctors and nurses under real-world conditions. We believe that the availability of our mHealth intervention will encourage Nigerian medical doctors and nurses who are experiencing the ongoing pandemic-related psychological distress to use a platform that does not necessitate their need to seek face-to-face consultation with a psychiatrist or other mental health specialists [5]. Any technological development in the context of primary and public health can potentially positively impact disease control, thereby minimizing complications and treatment costs [46,47]. However, some patients and providers may hesitate to use mHealth interventions due to low levels of health literacy, low familiarity with mobile apps and technology, or reduced access to mobile and internet facilities.

We are hopeful that our mHealth intervention will encourage a positive help-seeking attitude among Nigerian medical doctors and nurses experiencing COVID-19–related psychological distress.

## Abbreviations

LASUTH: Lagos State University Teaching Hospital
mHealth: mobile health
OAUTHC: Obafemi Awolowo University Teaching Hospitals Complex
SPIRIT: Standard Protocol Item: Recommendations for International Trials

## Data Availability

The data sets generated during this study are available from the corresponding author on reasonable request.
